# Mortality of Philadelphia for April, May and June, 1855; Collated from the Health Office Record

**Published:** 1855-08

**Authors:** Wilson Jewell


*Mortality of Philadelphia for April, May and June,* 1855; *collated from the Health Office Record.* By WILSON JEWELL, M. D.

Within the quarter of the year ending June 30th, including a period of ninety days, there were 2374 deaths returned to the Health Office, averaging 26 per day. This number is less by ninety-five than was returned for the same period in 1854. Thus far, according to the tabular statement of deaths for the first six months, the city has enjoyed a greater degree of health than during the first half of last year; the mortality constituting the evidence.

The deaths from recorded diseases alone, have amounted to 1922. The remaining deaths, in all 452, are charged to external causes, old age, still-born, debility and unknown.

The excess of deaths in the male sex, amounts to 6.68 per cent. The males number 1268; the females 1106.

The deaths within the first year of life, not including the still-born, amounted to 512. Within the fifth year 920. Within the tenth year 1031, and within the twentieth 1129. According to these statistics, it appears that 32.62 per cent. of the mortality of our population, took place within the first decade of life, and 33.58 per cent. within the second. These calculations exclude still-born children, which amounted to one hundred and fifty.

The greatest mortality during the quarter, has been from those diseases of an inflammatory type, arranged under the head of Organs of Respiration, amounting to 624; five sevenths of which, or 543 are charged to Consumption of the Lungs, Inflammation of the Lungs and Inflammation of the Bronchia.

The deaths from Consumption of the Lungs, Still born, and Inflammation of the Lungs, each of which exceed one hundred, together with Debility which wants one of making one hundred, in the aggregate 868, constitute more than one third, or 43.36 per cent. of all the deaths for the quarter.

The decrease in the deaths from bowel complaints, at this season of the year, when compared with those in the second or like quarter of last year, presents a sensible difference. Last year in the second quarter the deaths from Cholera, Cholera Morbus, Cholera Infantum, Diarrhœa and Dysentery, amounted to 173; whereas, the second quarter of the present year furnishes only 78—less 38.33 per cent.

The deaths from Small Pox, show a large increase over those for the same quarter of 1854; equal to 52.94 per cent. For the first half of the present year they have amounted to 93; more than double the number recorded for the whole of 1854.

Hooping Cough presents a considerable falling off. Last year the second quarter gave 45; this year 10; a diminution of three fourths.

The deaths from Measles likewise have declined 55 per cent. when compared with those for the same quarter of last year.

The deaths from Epidemic diseases exhibit a falling off of 15.80 per cent. contrasted with those of similar months in 1854.

The deaths from Still Born have increased 8.30 per cent. over those for the same months in last year.

Three of the deaths during the quarter were Centennarians, one of whom had reached her 115th year.

The Alms-house and the colored population yielded each about 8 per cent. of the deaths for the quarter.

On the whole, therefore, it may be safely inferred that the past six months have been more than usually healthy.

TABLE NO. I.

*Deaths for the second quarter of 1855 classified.*

| | Ap. | May | June | Male. A. | Male. M. | Male. J. | Female. A. | Female. M. | Female. J. | Total. |
|---|---|---|---|---|---|---|---|---|---|---|
| 1 *Endemic & Contagious diseases* Zymotic or Epidemic | 113 | 130 | 124 | 50 | 69 | 69 | 63 | 61 | 55 | 367 |
| 2 *Uncertain or general seat,* Sporadic diseases | 102 | 120 | 89 | 51 | 69 | 50 | 51 | 51 | 39 | 311 |
| 3 Nervous system | 149 | 167 | 146 | 82 | 90 | 77 | 67 | 77 | 69 | 462 |
| 4 Organs of Respiration | 222 | 271 | 131 | 111 | 143 | 73 | 111 | 128 | 58 | 624 |
| 5 " Circulation | 16 | 32 | 30 | 9 | 22 | 13 | 7 | 10 | 17 | 78 |
| 6 Digestive organs | 41 | 53 | 43 | 20 | 27 | 19 | 21 | 26 | 24 | 137 |
| 7 Urinary " | 6 | 2 | 6 | 3 | 1 | 5 | 3 | 1 | 1 | 14 |
| 8 Organs of Generation | 4 | 7 | 4 | | 1 | | 4 | 6 | 4 | 15 |
| 9 " Locomotion | | 3 | 2 | | 1 | 1 | | 2 | 1 | 5 |
| 10 Integumentary system | 3 | 1 | 4 | 1 | 1 | 2 | 2 | | 2 | 8 |
| 11 Old age | 17 | 20 | 10 | 7 | .9 | 3 | 10 | 11 | 7 | 47 |
| 12 External causes | 37 | 33 | 28 | 26 | 25 | 27 | 11 | 8 | 1 | 98 |
| Still Born | 42 | 48 | 60 | 27 | 24 | 33 | 15 | 24 | 27 | 150 |
| Unknown | 18 | 22 | 18 | 11 | 9 | 7 | 7 | 13 | 11 | 58 |
| | 770 | 909 | 695 | 398 | 491 | 379 | 372 | 418 | 316 | 2374 |

### TABLE NO. 2.

1. *Endemic and Contagious Diseases—Zymotic or Epidemic.*

| | Male. | Female. | Under 1 yr. | 1 to 2. | 2 to 5. | 5 to 10. | 10 to 15. | 15 to 20. | 20 to 30. | 30 to 40. | 40 to 50. | 50 to 60. | 60 to 70. | 70 to 80. | 80 to 90. | 90 to 100. | 100 to 110. | Total. |
|---|---|---|---|---|---|---|---|---|---|---|---|---|---|---|---|---|---|---|
| Cholera infantum | 13 | 6 | 14 | 3 | 2 | | | | | | | | | | | | | 19 |
| " morbus | 4 | 3 | 1 | | | | | | 1 | 1 | 1 | 1 | 1 | 1 | | | | 7 |
| Croup | 30 | 21 | 5 | 16 | 28 | 2 | | | | | | | | | | | | 51 |
| Diarrhœa | 13 | 12 | 6 | 4 | | | | | 2 | | 2 | 4 | 2 | 3 | | 2 | | 25 |
| Dysentery | 13 | 14 | 4 | 4 | 2 | | | | 3 | 7 | 3 | 1 | 2 | 1 | | | | 27 |
| Erysipelas | 14 | 15 | 13 | 2 | 1 | | | | 2 | | 4 | 2 | 1 | 2 | 2 | | | 29 |
| Fever, | 1 | 1 | | 2 | | | | | | | | | | | | | | 2 |
| " Bilious | 6 | 6 | | 1 | .1 | | | | 2 | 1 | | 3 | | 4 | | | | 12 |
| " Congestive | | 1 | | | | | | | | | | 1 | | | | | | 1 |
| " Intermittent | | 1 | | 1 | | | | | | | | | | | | | | 1 |
| " Puerperal | | 20 | | | | | | 1 | 16 | 2 | 1 | | | | | | | 20 |
| " Remittent | 2 | 3 | 1 | 1 | 1 | | | | 1 | 1 | | | | | | | | 5 |
| " Scarlet | 18 | 17 | 4 | 8 | 16 | 6 | 1 | | | | | | | | | | | 35 |
| " Typhoid | 21 | 18 | | | 3 | 4 | 2 | 5 | 6 | 5 | 7 | 3 | 3 | 1 | | | | 39 |
| " Typhus | 8 | 5 | | | | | | 1 | 6 | 1 | 3 | 2 | | | | | | 13 |
| Hooping Cough | 8 | 2 | 8 | 2 | | | | | | | | | | | | | | 10 |
| Influenza | 1 | 2 | | | | | | | | | 1 | | 1 | 1 | | | | 3 |
| Measles | 4 | 5 | 4 | | 5 | | | | | | | | | | | | | 9 |
| Small Pox | 30 | 22 | 10 | 10 | 16 | 6 | 2 | 1 | 5 | 2 | | | | | | | | 52 |
| Syphilis | | 1 | | 1 | | | | | | | | | | | | | | 1 |
| " secondary | | 1 | | | | | | | 1 | | | | | | | | | 1 |
| Varioloid | 2 | 3 | 1 | | 3 | 1 | | | | | | | | | | | | 5 |
| | 188 | 179 | 71 | 55 | 8 | 19 | 5 | 18 | 45 | 20 | 22 | 17 | 10 | 13 | 2 | 2 | | 367 |

2. *Uncertain or General Seat—Sporadic Diseases.*

| | Male. | Female. | Under 1 yr. | 1 to 2. | 2 to 5. | 5 to 10. | 10 to 15. | 15 to 20. | 20 to 30. | 30 to 40. | 40 to 50. | 50 to 60. | 60 to 70. | 70 to 80. | 80 to 90. | 90 to 100. | 100 to 110. | Total. |
|---|---|---|---|---|---|---|---|---|---|---|---|---|---|---|---|---|---|---|
| Abscess | 2 | 1 | 1 | | 1 | | | 1 | | | | | | | | | | 3 |
| " Breast | | 1 | | | | | | | | 1 | | | | | | | | 1 |
| " Lumbar | | 1 | | | | 1 | | | | | | | | | | | | 1 |
| " Psoas | | 1 | | | | | | | | | | | 1 | | | | | 1 |
| " Thigh | 1 | | | | | | | | | | 1 | | | | | | | 1 |
| Atrophy | 1 | 3 | 3 | | 1 | | | | | | | | | | | | | 4 |
| Cachexia | 1 | | 1 | | | | | | | | | | | | | | | 1 |
| Cancer | 4 | 1 | | | | | | | | 1 | | 2 | 1 | 1 | | | | 5 |
| " Breast | | 3 | | | | | | | | 2 | | | | 1 | | | | 3 |
| " Face | 1 | | | | | | | | | | | | | 1 | | | | 1 |
| Cyanosis | 6 | 4 | 9 | 1 | | | | | | | | | | | | | | 10 |
| Debility | 53 | 46 | 38 | | 1 | 1 | | 1 | 2 | 9 | 7 | 11 | 11 | 10 | 6 | 2 | | 99 |
| Dropsy | 15 | 14 | | | 1 | 1 | | | 4 | 2 | 2 | 6 | 7 | 3 | 2 | 1 | | 29 |
| Gangrene | 1 | 1 | | | 1 | | | | | | 1 | | | | | | | 2 |
| Hemorrhage | 4 | | 1 | | | | | | 1 | 1 | | | | | | 1 | | 4 |
| Inflammation | 1 | 1 | | | 1 | 1 | | | | | | | | | | | | 2 |
| Inanition | 13 | 10 | 17 | 2 | | | | | | 1 | 1 | 2 | | | | | | 23 |
| Inflammation of Throat | 1 | 3 | 2 | | | | | | | 1 | | | | 1 | | | | 4 |
| Malformation | 7 | 3 | 10 | | | | | | | | | | | | | | | 10 |
| Marasmus | 40 | 32 | 46 | 8 | 10 | 1 | | | 1 | 3 | | | | 1 | 2 | | | 72 |
| Mortification | 2 | 2 | | | | | | | | | 1 | 1 | 1 | 1 | | | | 4 |
| Scrofula | 8 | 6 | 3 | 1 | 1 | 4 | 1 | 2 | 1 | 1 | | | | | | | | 14 |
| Schirrhus | | 1 | | | | | | | | | | 1 | | | | | | 1 |
| Tumour of Abdomen | | 2 | | | | | | | | 1 | | 1 | | | | | | 2 |
| Tabes Mesenterica | 5 | 3 | 2 | 2 | 1 | 1 | | | | 1 | 1 | | | | | | | 8 |
| Ulcer | 1 | | | | | | | | | | | 1 | | | | | | 1 |
| Ulcer of Throat | 3 | 2 | 1 | | 1 | 3 | | | | | | | | | | | | 5 |
| | 170 | 141 | 134 | 14 | 19 | 13 | 1 | 4 | 9 | 24 | 14 | 25 | 21 | 19 | 10 | 4 | | 311 |

3. *Nervous Diseases.*

| | Male. | Female. | Under 1 yr. | 1 to 2. | 2 to 5. | 5 to 10. | 10 to 15. | 15 to 20. | 20 to 30. | 30 to 40. | 40 to 50. | 50 to 60. | 60 to 70. | 70 to 80. | 80 to 90. | 90 to 100. | 100 to 110. | Total. |
|---|---|---|---|---|---|---|---|---|---|---|---|---|---|---|---|---|---|---|
| Apoplexy | 17 | 18 | | | | | | | 2 | 9 | 4 | 2 | 9 | 6 | 3 | | | 35 |
| Concussion of Brain | 1 | | | | | | | | | | 1 | | | | | | | 1 |
| Congestion " | 24 | 10 | 9 | 6 | 3 | 6 | 1 | | 2 | | 4 | 2 | | 1 | | | | 34 |
| Compression " | 2 | | | | | | | | 1 | | | 1 | | | | | | 2 |
| Convulsions | 81 | 70 | 99 | 20 | 20 | 6 | 1 | 1 | 1 | 1 | 2 | | | | | | | 151 |
| Disease of Brain | 16 | 9 | 11 | 3 | 1 | 1 | 2 | | 2 | 3 | 1 | 1 | | | | | | 25 |
| Dropsy " | 28 | 27 | 20 | 16 | 10 | 5 | 1 | | | 1 | | | 1 | 1 | | | | 55 |
| Effusion " | 5 | 9 | 1 | 1 | 6 | 3 | 1 | | 1 | | 1 | | | | | | | 14 |
| Epilepsy | 3 | 4 | 1 | 1 | | | | | | 1 | 1 | 2 | 1 | | | | | 7 |
| Inflammation of Brain | 38 | 40 | 21 | 15 | 18 | 13 | 1 | 2 | | 5 | 1 | 1 | | 1 | | | | 78 |
| Insanity | | 1 | | | | | | | | | | 1 | | | | | | 1 |
| Mania | 5 | 3 | | | | | | | 1 | 3 | 1 | 3 | | | | | | 8 |
| " a Potu | 4 | 1 | | | | | | | 1 | | 4 | | | | | | | 5 |
| Palsy | 17 | 16 | | 1 | | | 3 | | 2 | 3 | 2 | 3 | 6 | 7 | 6 | | | 33 |
| Softening of Brain | 3 | 3 | | | 1 | 1 | | | | | | 1 | 2 | 1 | | | | 6 |
| Tetanus | 2 | 3 | | | 1 | | | 3 | 12 | | | | | | | | | 5 |
| Trismus | 2 | | 2 | | | | | | | | | | | | | | | 2 |
| | 248 | 214 | 164 | 63 | 60 | 35 | 10 | 6 | 14 | 26 | 22 | 17 | 19 | 17 | 9 | | | 462 |

### 4. *Organs of Respiration.*

| | Male. | Female. | Under 1 yr. | 1 to 2. | 2 to 5. | 5 to 10. | 10 to 15. | 15 to 20. | 20 to 30. | 30 to 40. | 40 to 50. | 50 to 60. | 60 to 70. | 70 to 80. | 80 to 90. | 90 to 100. | 100 to 110. | Total. |
|---|---|---|---|---|---|---|---|---|---|---|---|---|---|---|---|---|---|---|
| Abscess of Lungs | 2 | 3 | 1 | | | 1 | | | 1 | 1 | 1 | | | | | | | 5 |
| Asthma | 1 | 4 | 2 | 1 | | | | | | | 1 | 1 | | | | | | 5 |
| Congestion of Lungs | 12 | 7 | 4 | 2 | | | | | 2 | 1 | 2 | 2 | 2 | 4 | | | | 19 |
| Consumption " | 167 | 164 | 13 | 3 | 8 | 6 | 6 | 25 | 102 | 62 | 50 | 25 | 20 | 7 | 4 | | | 331 |
| Disease of " | | 10 | 2 | 2 | 2 | | | | | 2 | 1 | | 1 | | | | | 10 |
| Dropsy of Chest | 9 | 6 | | 3 | 2 | 1 | | | | | 3 | 1 | 1 | 3 | 1 | | | 15 |
| Effusion of Lungs | 2 | 3 | | | | | | | 1 | 1 | | 1 | 1 | 1 | | | | 5 |
| Gangrene " | | 1 | | | | | | | | 1 | | | | | | | | 1 |
| Hemorrhage of Lungs | 5 | | | | | | | | 2 | 1 | 1 | 1 | | | | | | 5 |
| Inflamm'n of Bronchiæ | 46 | 29 | 35 | 14 | 6 | 7 | | 1 | 2 | 2 | 1 | 2 | 1 | 3 | 1 | | | 75 |
| " Lungs | 75 | 62 | 29 | 19 | 11 | 6 | 2 | 3 | 14 | 17 | 10 | 12 | 8 | 3 | 3 | | | 137 |
| " Larynx | 3 | 3 | 1 | 2 | 2 | | | | 1 | | | | | | | | | 6 |
| " Pleura | 5 | 3 | | 1 | 1 | | | 1 | 1 | | 1 | 1 | 2 | | | | | 8 |
| " Trachea | | 1 | | | 1 | | | | | | | | | | | | | 1 |
| " Tonsils | | 1 | | 1 | | | | | | | | | | | | | | 1 |
| | 327 | 297 | 87 | 48 | 33 | 21 | 8 | 30 | 126 | 88 | 71 | 46 | 36 | 21 | 9 | | | 624 |

### 5. *Organs of Circulation.*

| | Male. | Female. | Under 1 yr. | 1 to 2. | 2 to 5. | 5 to 10. | 10 to 15. | 15 to 20. | 20 to 30. | 30 to 40. | 40 to 50. | 50 to 60. | 60 to 70. | 70 to 80. | 80 to 90. | 90 to 100. | 100 to 110. | Total. |
|---|---|---|---|---|---|---|---|---|---|---|---|---|---|---|---|---|---|---|
| Anæmia | 3 | 2 | 2 | | | 2 | | | | | | 1 | | | | | | 5 |
| Aneurism | 1 | | | | | | | | | | 1 | | | | | | | 1 |
| Angina Pectoris | | 1 | | | | | | | | | 1 | | | | | | | 1 |
| Disease of Heart | 24 | 26 | 2 | | 1 | 4 | 3 | 6 | 8 | 3 | 4 | 10 | 4 | 4 | 1 | | | 50 |
| Dropsy " | 5 | | | | 1 | 1 | | | | 1 | | 1 | | 1 | | | | 5 |
| Effusion " | | 1 | | | | | | | | | | | | 1 | | | | 1 |
| Enlargem't " | 6 | 3 | | | | | 1 | | 4 | 3 | | 1 | | | | | | 9 |
| Inflammat'n " | 4 | | | | | | | 1 | 2 | | 1 | | | | | | | 4 |
| Rupture of Aorta | | 1 | | | | | | | | | | 1 | | | | | | 1 |
| " Heart | 1 | | | | | | | | | 1 | | | | | | | | 1 |
| | 44 | 34 | 4 | | 2 | 7 | 4 | 7 | 14 | 8 | 7 | 14 | 4 | 6 | 1 | | | 78 |

### 6. *Digestive Organs.*

| | Male. | Female. | Under 1 yr. | 1 to 2. | 2 to 5. | 5 to 10. | 10 to 15. | 15 to 20. | 20 to 30. | 30 to 40. | 40 to 50. | 50 to 60. | 60 to 70. | 70 to 80. | 80 to 90. | 90 to 100. | 100 to 110. | Total. |
|---|---|---|---|---|---|---|---|---|---|---|---|---|---|---|---|---|---|---|
| Abscess of Liver | 1 | | | | | | | | | | 1 | | | | | | | 1 |
| Apthæ | 1 | | 1 | | | | | | | | | | | | | | | 1 |
| Cancer of Bowels | | 1 | | | | | | | | | | 1 | | | | | | 1 |
| " Liver | | 1 | | | | | | | | | 1 | | | | | | | 1 |
| " Stomach | 1 | 2 | | | | | | | | | | | 2 | 1 | | | | 3 |
| " Rectum | | 1 | | | | | | | | | 1 | | | | | | | 1 |
| Cancrum Oris | 2 | | | 1 | 1 | | | | | | | | | | | | | 2 |
| Cirrhosis of Liver | 3 | | | | | | | | 1 | | 1 | 1 | | | | | | 3 |
| Congestion of " | 2 | | | | | | | | 1 | | | | 1 | | | | | 2 |
| Consumption of Bowels | | 2 | | | | 1 | | | | | | | 1 | | | | | 2 |
| Colic | 1 | 1 | | | | | | | 1 | | | 1 | | | | | | 2 |
| Disease of Bowels | 2 | 4 | 1 | | 1 | | | | 1 | 2 | | | | 1 | | | | 6 |
| " Liver | 5 | 3 | 1 | | | | | | | 2 | 3 | 1 | 1 | | | | | 8 |
| " Stomach | | 1 | | | | | | | | | 1 | | | | | | | 1 |
| Dyspepsia | 1 | | | | | | | | | | | 1 | | | | | | 1 |
| Dropsy of Abdomen | 3 | 4 | | | | | 1 | | 1 | 1 | 1 | 3 | | | | | | 7 |
| Hemorrhage Umbilicus | 1 | 1 | 2 | | | | | | | | | | | | | | | 2 |
| Hernia Strangulated | 1 | 1 | | | | | | | | 1 | | | | 1 | | | | 2 |
| Inflammation of Bowels | 15 | 10 | 7 | 3 | 2 | 1 | | 1 | 4 | 1 | 4 | 1 | | 1 | | | | 25 |
| " Colon | | 1 | | 1 | | | | | | | | | | | | | | 1 |
| " Liver | 4 | 5 | 1 | | | | | | | 2 | | 1 | 3 | | 2 | | | 9 |
| " Peritoneum | 5 | 12 | 1 | | 1 | 1 | 1 | 1 | 3 | 1 | 2 | 4 | 1 | | 1 | | | 17 |
| " Stomach | 1 | 5 | 1 | | | | | | | 1 | 1 | | 2 | 1 | | | | 6 |
| " Stom. & Bowels | 9 | 2 | 4 | 1 | | 1 | | | 1 | | 2 | | 1 | 1 | | | | 11 |
| " Spleen | 1 | | | | | 1 | | | | | | | | | | | | 1 |
| Intussusception | 1 | 2 | 1 | | | | | | 1 | | | | | 1 | | | | 3 |
| Jaundice | | 2 | | | | | | | | | | | 1 | 1 | | | | 2 |
| Mortification of Bowels | 1 | | 1 | | | | | | | | | | | | | | | 1 |
| Obstruction " | 1 | 2 | | | | | | | 1 | 1 | 1 | | | | | | | 3 |
| Stricture of Rectum | 1 | | | | | | | 1 | | | | | | | | | | 1 |
| Teething | | 3 | 1 | 1 | 1 | | | | | | | | | | | | | 3 |
| Ulceration of Bowels | | 2 | | | | | | | 1 | | | | | 1 | | | | 2 |
| " Stomach | 2 | | | | 1 | | | | | 1 | | | | | | | | 2 |
| Worms | 1 | 3 | 1 | 1 | 2 | | | | | | | | | | | | | 4 |
| | 66 | 71 | 23 | 8 | 9 | 5 | 2 | 3 | 16 | 13 | 19 | 14 | 13 | 9 | 3 | | | 137 |

### 7. *Urinary Organs.*

| | Male. | Female. | Under 1 yr. | 1 to 2. | 2 to 5. | 5 to 10. | 10 to 15. | 15 to 20. | 20 to 30. | 30 to 40. | 40 to 50. | 50 to 60. | 60 to 70. | 70 to 80. | 80 to 90. | 90 to 100. | 100 to 110. | Total. |
|---|---|---|---|---|---|---|---|---|---|---|---|---|---|---|---|---|---|---|
| Albuminuria | 3 | | | | | | | | 1 | | 2 | | | | | | | 3 |
| Cancer of Bladder | | 1 | | | | | | | | | | 1 | | | | | | 1 |
| " Kidneys | | 1 | | | | | | | | | | | | | 1 | | | 1 |
| Diabetes | 1 | | | | | | | | | 1 | | | | | | | | 1 |
| Disease of Kidneys | 1 | 1 | | | | | | | 2 | | | | | | | | | 2 |
| Inflamed Bladder | 1 | 1 | | | | | | | 1 | 1 | | | | | | | | 2 |
| " Kidneys | | 1 | | | | | | | | | 1 | | | | | | | 1 |
| Rupture of Bladder | 1 | | | | | | | | | | | 1 | | | | | | 1 |
| Stone in " | 1 | | | | | | | | | | | | 1 | | | | | 1 |
| Uræmia | 1 | | | | | | | | | | | | | 1 | | | | 1 |
| | 9 | 5 | | | | | | | 4 | 2 | 3 | 2 | 1 | 1 | 1 | | | 14 |

### 8. *Organs of Generation.*

| | Male. | Female. | Under 1 yr. | 1 to 2. | 2 to 5. | 5 to 10. | 10 to 15. | 15 to 20. | 20 to 30. | 30 to 40. | 40 to 50. | 50 to 60. | 60 to 70. | 70 to 80. | 80 to 90. | 90 to 100. | 100 to 110. | Total. |
|---|---|---|---|---|---|---|---|---|---|---|---|---|---|---|---|---|---|---|
| Cancer of Uterus | | 5 | | | | | | | | 1 | 1 | 2 | | 1 | | | | 5 |
| Disease of Prostate Gland | 1 | | | | | | | | | | | | | 1 | | | | 1 |
| " Uterus | | 1 | | | | | | | | | | | 1 | | | | | 1 |
| Dropsy of Ovaries | | 1 | | | | | | | | | | | 1 | | | | | 1 |
| Hemorrhage of Uterus | | 3 | | | | | | | 1 | 1 | 1 | | | | | | | 3 |
| Inflammation " | | 2 | | | | | | 1 | 1 | | | | | | | | | 2 |
| Phlegmasia Dolens | | 1 | | | | | | | 1 | | | | | | | | | 1 |
| Puerperal Convulsions | | 1 | | | | | | | 1 | | | | | | | | | 1 |
| | 1 | 14 | | 1 | | | | 1 | 4 | 2 | 2 | 2 | 2 | 2 | | | | 15 |

### 9. *Organs of Locomotion.*

| | Male. | Female. | Under 1 yr. | 1 to 2. | 2 to 5. | 5 to 10. | 10 to 15. | 15 to 20. | 20 to 30. | 30 to 40. | 40 to 50. | 50 to 60. | 60 to 70. | 70 to 80. | 80 to 90. | 90 to 100. | 100 to 110. | Total. |
|---|---|---|---|---|---|---|---|---|---|---|---|---|---|---|---|---|---|---|
| Inflammation of Spine | 1 | | | | 1 | | | | | | | | | | | | | 1 |
| Rheumatism | 1 | 2 | | | | | | | 1 | | 1 | 1 | | | | | | 3 |
| Spina Bifida | | 1 | 1 | | | | | | | | | | | | | | | 1 |
| | 2 | 3 | 1 | | 1 | | | | 1 | | 1 | 1 | | | | | | 5 |

### 10. *Integumentary System.*

| | Male. | Female. | Under 1 yr. | 1 to 2. | 2 to 5. | 5 to 10. | 10 to 15. | 15 to 20. | 20 to 30. | 30 to 40. | 40 to 50. | 50 to 60. | 60 to 70. | 70 to 80. | 80 to 90. | 90 to 100. | 100 to 110. | 110 to 120. | Total. |
|---|---|---|---|---|---|---|---|---|---|---|---|---|---|---|---|---|---|---|---|
| Disease of Skin | | 1 | | 1 | | | | | | | | | | | | | | | 1 |
| Purpura Hemorrhagica | 3 | 2 | 3 | | 1 | | | | 1 | | | | | | | | | | 5 |
| Rupia | 1 | | | | 1 | | | | | | | | | | | | | | 1 |
| Scurvy | 1 | | | | | | | | | 1 | | | | | | | | | 1 |
| | 5 | 3 | 3 | 1 | 2 | | | | 1 | 1 | | | | | | | | | 8 |

### 11. *Old Age.*

| | | | | | | | | | | | | | | | | | | | |
|---|---|---|---|---|---|---|---|---|---|---|---|---|---|---|---|---|---|---|---|
| Old Age | 19 | 28 | | | | | | | | | | | 6 | 16 | 21 | 1 | 2 | 1 | 47 |

12. *External Causes.*

| | Male. | Female. | Under 1 yr. | 1 to 2. | 2 to 5. | 5 to 10. | 10 to 15. | 15 to 20. | 20 to 30. | 30 to 40. | 40 to 50. | 50 to 60. | 60 to 70. | 70 to 80. | 80 to 90. | 90 to 100. | 100 to 110. | Total. |
|---|---|---|---|---|---|---|---|---|---|---|---|---|---|---|---|---|---|---|
| Asphyxia | 3 | 3 | 4 | | | | | | | 1 | 1 | | | | | | | 6 |
| Burns | 4 | 5 | | 3 | 3 | 1 | 1 | | 1 | | | | | | | | | 9 |
| Casualties | 19 | 3 | | 2 | | 2 | 2 | 2 | 4 | 5 | 1 | 2 | 1 | 1 | | | | 22 |
| Drowned | 33 | 1 | | | 1 | 7 | 2 | 2 | 2 | 7 | 9 | 3 | 1 | | | | | 34 |
| Excessive Heat | 1 | | | | | | | | | | 1 | | | | | | | 1 |
| Fracture | 1 | | | | | | | | 1 | | | | | | | | | 1 |
| Exhaustion | | 1 | | | | | | | | | | | | 1 | | | | 1 |
| Intemperance | 9 | 4 | | | | | | | 3 | 5 | 2 | 1 | 1 | 1 | | | | 13 |
| Strangulation | | 1 | | | | | | | | | | 1 | | | | | | 1 |
| Suffocation | 3 | 1 | 1 | | 1 | 1 | | | 1 | | | | | | | | | 4 |
| Suicide | 5 | | | | | | | | 1 | | | 3 | | 1 | | | | 5 |
| Violence | | 1 | | | | | | | 1 | | | | | | | | | 1 |
| | 78 | 20 | 5 | 5 | 5 | 11 | 5 | 4 | 14 | 18 | 14 | 10 | 3 | 4 | | | | 98 |
| Still Born | 84 | 66 | | | | | | | | | | | | | | | | 150 |
| Unknown | 27 | 31 | 20 | 2 | 3 | | | | 6 | 8 | 7 | 3 | 3 | 6 | | | | 58 |

TABLE NO. 3.

*Deaths for the Second Quarter of* 1855, *at sixteen distinct periods of life.*

| | |
|---|---|
| Under 1 year, . . . . . . . | 512 |
| 1 to 2 . . . . . . . | 196 |
| 2 to 5 . . . . . . . | 212 |
| 5 to 10 . . . . . . . | 111 |
| 10 to 15 . . . . . . . | 35 |
| 15 to 20 . . . . . . . | 63 |
| 20 to 30 . . . . . . . | 254 |
| 30 to 40 . . . . . . . | 210 |
| 40 to 50 . . . . . . . | 182 |
| 50 to 60 . . . . . . . | 151 |
| 60 to 70 . . . . . . . | 118 |
| 70 to 80 . . . . . . . | 114 |
| 80 to 90 . . . . . . . | 56 |
| 90 to 100 . . . . . . . | 7 |
| 100 to 110 . . . . . . . | 2 |
| 110 to 120 . . . . . . . | 1 |
| | 2224 |
| Still Born . . . . . . . | 150 |
| Total, | 2374 |

Embraced within the above table were 190 from the Blockley Alms House; 172 Blacks, and 24 from the country, as follows:

| | April, | May. | June. | Total. |
|---|---|---|---|---|
| Almshouse, . . | 65 | 73 | 52 | 190 |
| Blacks, . . . | 70 | 61 | 41 | 172 |
| Country, . . | 5 | 8 | 11 | 24 |
| Total, | | | | 386 |

